# Malignant tumors in tuberous sclerosis complex: a case report and review of the literature

**DOI:** 10.1186/s12920-024-01913-8

**Published:** 2024-05-27

**Authors:** Cassie Liu, Subodh M. Lele, Martin H. Goodenberger, Gwendolyn M. Reiser, Andrew J. Christiansen, James C. Padussis

**Affiliations:** 1 Disivion of Surgical Oncology, Department of Surgery, Fred and Pamela Buffett Cancer Center, University of Nebraska Medical Center, Omaha, NE USA; 2 Department of Pathology and Microbiology, University of Nebraska Medical Center, Omaha, NE USA; 3 Department of Radiology, University of Nebraska Medical Center, Omaha, NE USA; 4 Genetic Medicine, Munroe-Meyer Institute, University of Nebraska Medical Center, Omaha, NE USA; 5 Division of Urologic Surgery, Department of Surgery, University of Nebraska Medical Center, Omaha, NE USA

**Keywords:** Tuberous sclerosis complex, *TSC1* gene mutation, Renal cell carcinoma, Pancreatic neuroendocrine tumor, Case report

## Abstract

**Background:**

Tuberous sclerosis complex (TSC) is a rare, autosomal dominant genetic disease that arises from *TSC1* or *TSC2* genetic mutations. These genetic mutations can induce the development of benign tumors in any organ system with significant clinical implications in morbidity and mortality. In rare instances, patients with TSC can have malignant tumors, including renal cell carcinoma (RCC) and pancreatic neuroendocrine tumor (PNET). It is considered a hereditary renal cancer syndrome despite the low incidence of RCC in TSC patients. TSC is typically diagnosed in prenatal and pediatric patients and frequently associated with neurocognitive disorders and seizures, which are often experienced early in life. However, penetrance and expressivity of *TSC* mutations are highly variable. Herein, we present a case report, with associated literature, to highlight that there exist undiagnosed adult patients with less penetrant features, whose clinical presentation may contain non-classical signs and symptoms, who have pathogenic *TSC* mutations.

**Case presentation:**

A 31-year-old female with past medical history of leiomyomas status post myomectomy presented to the emergency department for a hemorrhagic adnexal cyst. Imaging incidentally identified a renal mass suspicious for RCC. Out of concern for hereditary leiomyomatosis and renal cell carcinoma (HLRCC) syndrome, the mass was surgically removed and confirmed as RCC. Discussion with medical genetics ascertained a family history of kidney cancer and nephrectomy procedures and a patient history of ungual fibromas on the toes. Genetic testing for hereditary kidney cancer revealed a 5’UTR deletion in the *TSC1* gene, leading to a diagnosis of TSC. Following the diagnosis, dermatology found benign skin findings consistent with TSC. About six months after the incidental finding of RCC, a PNET in the pancreatic body/tail was incidentally found on chest CT imaging, which was removed and determined to be a well-differentiated PNET. Later, a brain MRI revealed two small cortical tubers, one in each frontal lobe, that were asymptomatic; the patient’s history and family history did not contain seizures or learning delays. The patient presently shows no evidence of recurrence or metastatic disease, and no additional malignant tumors have been identified.

**Conclusions:**

To our knowledge, this is the first report in the literature of a TSC patient without a history of neurocognitive disorders with RCC and PNET, both independently rare occurrences in TSC. The patient had a strong family history of renal disease, including RCC, and had several other clinical manifestations of TSC, including skin and brain findings. The incidental finding and surgical removal of RCC prompted the genetic evaluation and diagnosis of TSC, leading to a comparably late diagnosis for this patient. Reporting the broad spectrum of disease for TSC, including more malignant phenotypes such as the one seen in our patient, can help healthcare providers better identify patients who need genetic evaluation and additional medical care.

## Introduction

Tuberous sclerosis complex (TSC) is a rare, autosomal dominant genetic disease that can affect every organ system. Its penetrance and expressivity are highly variable, and clinical manifestations of TSC can differ widely even within families [1–3]. Diagnostic criteria, surveillance, and management are constantly evolving, last updated in 2021 [4]. The most common clinical presentations of TSC consist of benign tumors, including hamartomas, in the brain, kidneys, heart, lungs, skin, and eyes [2–5]. Despite their pathologically benign nature, these tumors can result in serious clinical consequences, including disability and death, and require lifetime surveillance and management [4].

TSC is only known to arise from either a pathogenic mutation in *TSC1* or *TSC2*; about 10–15% of TSC cases have been reported to arise without a known mutation in *TSC1* or *TSC2*, which may be a result of mosaicism [4]. Pathogenic *TSC1/2* gene variants are defined as mutations that either prevent protein function or functionally inactivate hamartin or tuberin [4]. There are presently thousands of *TSC1/2* variants reported, and the type and location of the mutation can affect phenotype [3, 6, 7]. Although TSC is a genetic disease, *TSC1/2* mutations are inherited only 30% of the time, with 70–90% cases inheriting a *TSC2* mutation [3, 8]. At a population level, mutations in *TSC2* are associated with more severe disease, including significant hamartomas, subependymal nodules, subependymal giant cell tumors, early-onset kidney disease, and pancreatic neuroendocrine tumors (PNET), and present with clinical symptoms at a younger age [2, 3, 5, 9]. Furthermore, *TSC2* lies directly adjacent to *PKD1*, the gene responsible for autosomal dominant polycystic kidney disease (ADPKD), and TSC patients can have large genetic deletions that span both genes in a disorder known as TSC2/PKD1 contiguous gene syndrome [10, 11].

Rarely, patients with TSC express malignant tumors. In a large, multinational cohort of TSC patients, malignancies occurred at a rate of 2.9%, at a median age of 31 years, most commonly in females, and in participants with a *TSC1* mutation [12]. Renal cell carcinoma (RCC) is considered the most common malignancy in TSC patients and presents in 2–5% of cases, usually bilaterally, more often in women, and at a younger age than the general population [2, 5, 13, 14]. PNET arise in 1–9% of cases and are thought to be associated with *TSC2* mutations [5, 15–19]. Both malignancies are included in the updated TSC surveillance guidelines, though neither contribute to TSC diagnostic criteria [4].

## Case presentation

A 31-year-old female with past medical history of leiomyomas status post myomectomy presented to the emergency department (ED) with 12 h of sudden 8/10 abdominal pain, worse on the right side than the left, unable to be relieved by ibuprofen. Abdomen and pelvis computed tomography (CT) revealed a large right adnexal cyst with hyperdense fluid within the right lower quadrant that extended up towards the liver, consistent with a hemorrhagic cyst in the setting of a negative pregnancy test. The CT also revealed an indeterminate 3 cm right renal mass concerning for RCC.

**Fig. 1:**
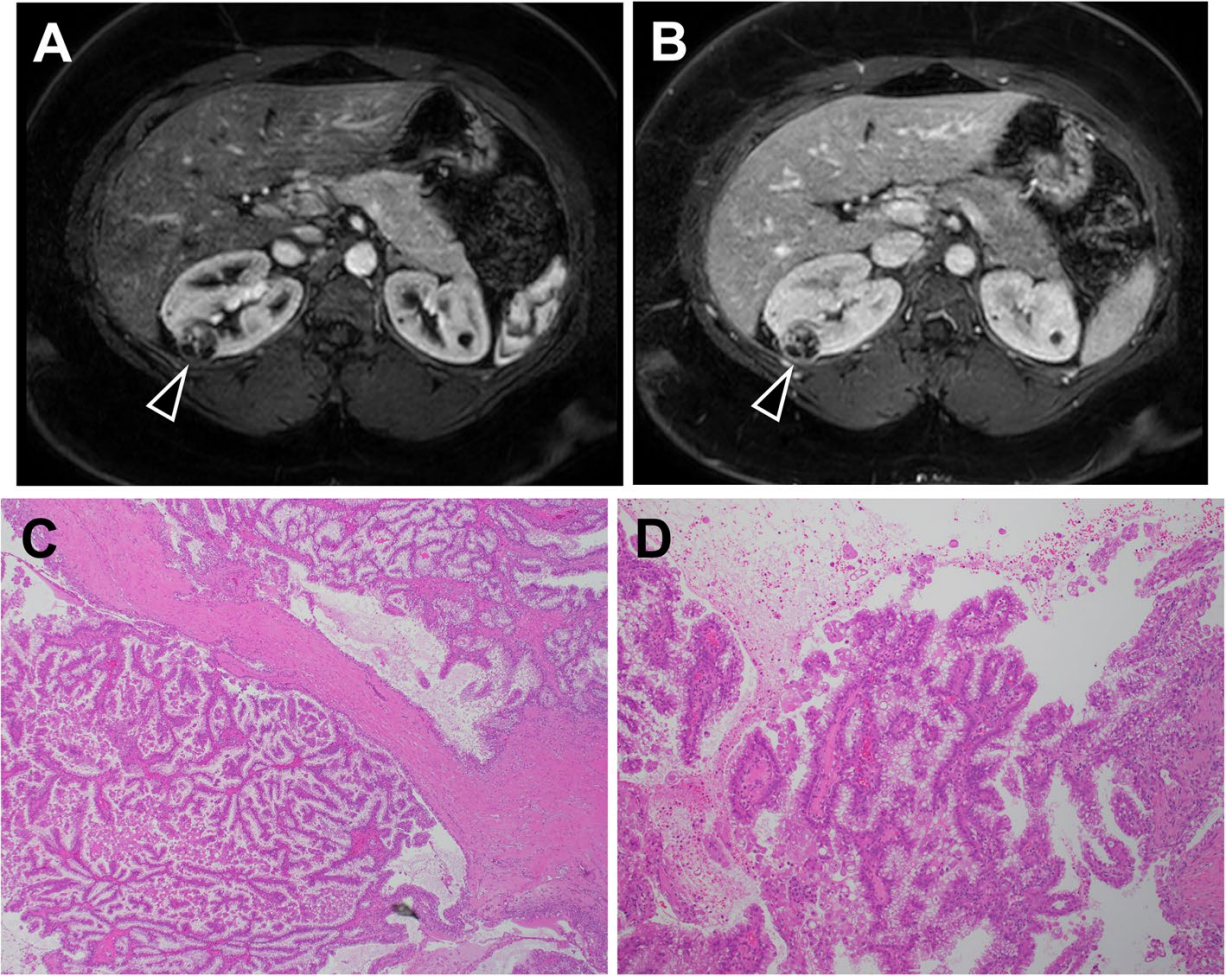
Renal cell carcinoma on (**A** & **B**) MR images and (**C** & **D**) pathology slides. Axial (**A**) arterial and (**B**) portal venous phase MR images of the abdomen. The right RCC, identified as a complex bosniak category IV 2.4 cm right renal cystic lesion on imaging, is delineated with the arrow. Hematoxylin and eosin stains of the RCC revealed (**C**) smooth muscle intersecting with tumor nests composed of cells with clear cytoplasm (original magnification x40) and (**D**) papillary-like tumor architecture lined by cells with clear cytoplasm without any antipodal nuclear arrangement (original magnification x100)

In a following outpatient appointment, the patient’s primary care provider discovered that the patient’s mother had renal cancer status post nephrectomy at age 42 and was presently on dialysis secondary to polycystic kidney disease (PCKD). The patient was promptly referred to urology, who was concerned for a potential diagnosis of hereditary leiomyomatosis and renal cell carcinoma (HLRCC) syndrome given history of uterine fibroids. Magnetic resonance imaging (MRI) of the abdomen and pelvis revealed a complex bosniak category IV 2.4 cm right renal cystic lesion, even more concerning for RCC (Fig. 1A-B).

Three months after ED presentation, the patient underwent a robotic partial nephrectomy without complications and was discharged the following day. Wide resection of the tumor was performed given concern for HLRCC. Pathology revealed a 2.5 cm RCC, tumor grade 2, with negative margins. AJCC stage was pT1a. The tumor had features that were not specific for any of the established types of RCC and, hence, was placed in the unclassified category. Specifically, the tumor exhibited cells with clear cytoplasm and low nuclear grade arranged in a papillary-like architecture with fibromyomatous-type intervening stroma (Fig. 1C-D). Immunostaining was positive for CK7, carbonic anhydrase 9, vimentin, and PAX8 and negative for several other markers. The patient was referred to medical genetics for further evaluation and testing.

Medical genetics uncovered a patient history of ungual fibromas removed from her toes and family history of kidney cancer in the patient’s mother at age 42 and maternal aunt at age 42. The patient also had a maternal first cousin who had their kidney removed at age 40 and a maternal aunt who had her kidney removed at an unknown age. Her mother, maternal aunt with kidney cancer, and maternal cousin all had hypopigmented patches of skin. Her mother may have had dental pits. Her paternal family history was limited but had no known cancer history. There was no known history of family members with other skin findings, seizures, learning delays, heart/lung/eye tumors, or complications. The patient did not have knowledge of the genetic status of family members. The patient’s ancestry was African American without Ashkenazi Jewish ancestry and consanguinity. RenalNext® test, a next-generation sequencing panel, was used to test 20 cancer susceptibility genes for hereditary kidney cancer: *BAP1*, *CHEK2*, *EPCAM*, *FH*, *FLCN*, *MET*, *MLH1*, *MSH2*, *MSH6*, *MITF*, *PMS2*, *PTEN*, *SDHA*, *SDHB*, *SDHC*, *SDHD*, *TP53*, *TSC1*, *TSC2*, and *VHL*. The test identified a 5’UTR deletion in the *TSC1* gene, a likely pathogenic variant. The remaining genes were negative for pathogenic variants and variants of uncertain significance. Later, dermatology identified on the patient additional benign skin findings, including small growths and hypopigmented regions.

**Fig. 2:**
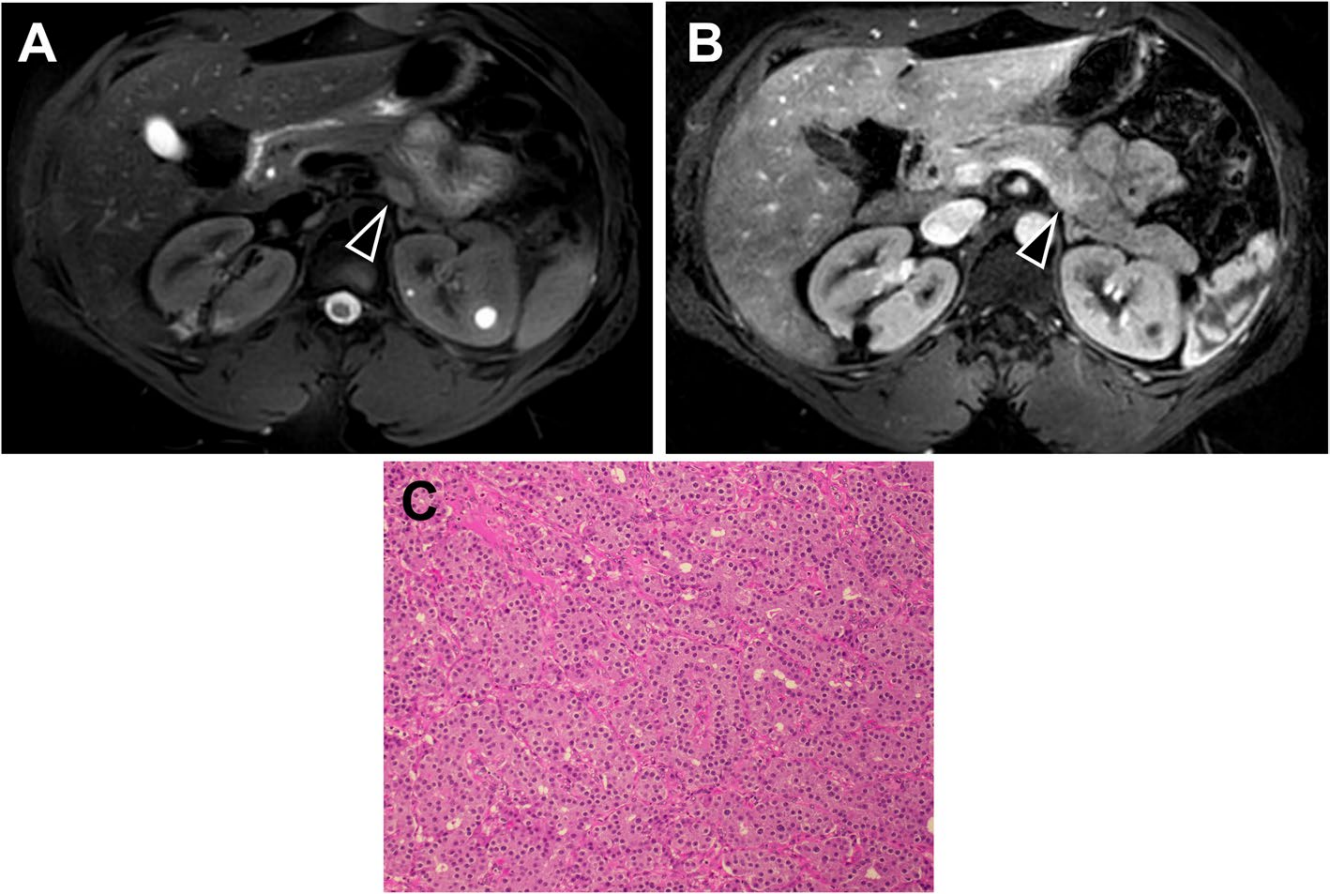
Pancreatic neuroendocrine tumor on (**A** & **B**) MRI magnetic resonance cholangiopancreatography and **(C)** pathology slides. Axial (**A**) weighted T2 and (**B**) early arterial phase MRCP images. The neuroendocrine tumor in the body/tail of the pancreas, identified as a 1.7 cm solid, enhancing lesion on imaging, is delineated with the arrow. (**C**) Hematoxylin and eosin stain of the PNET revealed clusters of monomorphic tumor cells with uniform nuclei and salt-and-pepper-type chromatin typical of this type of tumor (original magnification x200)

Three months after her partial nephrectomy, the patient received a chest CT to rule out concerns for pulmonary embolism, which incidentally revealed a possible enhancing pancreatic body/tail lesion that was indeterminate particularly given the history of RCC. A month later, MRI magnetic resonance cholangiopancreatography (MRCP) revealed a 1.7 cm solid, enhancing lesion involving the pancreatic body/tail, worrisome for metastasis in the setting of RCC (Fig. 2A-B). There was also a punctate 5 mm cystic lesion in the region of the pancreatic head. An echocardiogram was also performed at this time and was unremarkable. The patient promptly received an endoscopic ultrasound (EUS) with fine needle aspiration (FNA) biopsy of the pancreatic body/tail mass. Pathology of the biopsy revealed a well-differentiated neuroendocrine tumor, WHO grade 1. The patient was then referred to surgical oncology to evaluate for resection of the likely nonfunctional PNET.

About four months after the incidental PNET finding on CT, the patient underwent robot-assisted distal pancreatectomy and splenectomy. Pathology revealed a 1.8 cm well-differentiated neuroendocrine tumor, WHO grade 1 (Ki-67 < 1%) (Fig. 2C), and all 14 lymph nodes negative for metastatic tumor. AJCC stage was pT1N0. Her post-operative course was complicated by a grade B pancreatic fistula, as defined by ISGPS, which resolved with a drain placed by interventional radiology and antibiotics. CT of the chest, abdomen, and pelvis at the time noted scattered areas of bone sclerosis in the sacrum that was stable compared to previous known imaging without new destructive or aggressive osseus lesions. TSC patients often have sclerotic bone lesions, and the areas of bone sclerosis seen on CT could be related to her TSC.

**Fig. 3:**
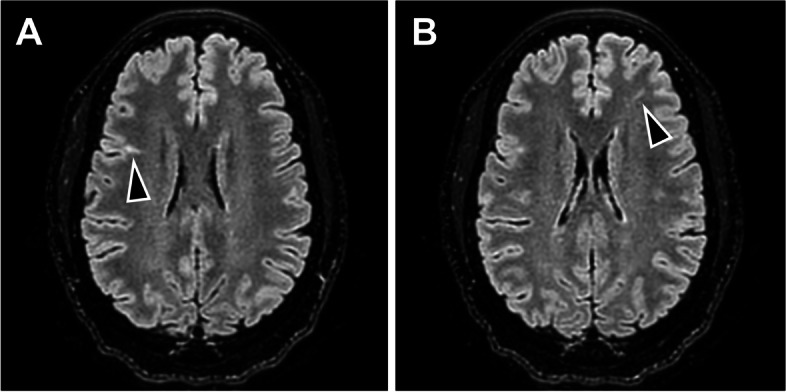
Tubers on MR images. (**A** & **B**) Two small, focal cortical tubers in the frontal lobes were identified on brain MRI. The patient did not exhibit clinical symptoms, including a history negative for seizures and learning delay. The tubers are delineated with the arrow

Neurology ordered a brain MRI a month following the distal pancreatectomy and splenectomy, which revealed two small, focal cortical dysplasias/tubers, one in each frontal lobe (Fig. 3), in addition to a very small focus in the right superior frontal lobe. The patient did not have a seizure history or present with neurocognitive dysfunction. A subsequent brain MRI one year later revealed no interval change, and neurology agreed to follow up if the patient becomes symptomatic in the future.

A year and a half following the distal pancreatectomy and splenectomy, the patient is currently negative for recurrence and metastatic disease of either RCC or PNET, and no additional malignant tumors have been identified.

## Discussion

TSC is generally a prenatal or pediatric diagnosis, with patients developing signs and symptoms as early as before the time of birth. In the case of our patient, the *TSC1* gene mutation was identified through an incidental finding of unifocal, unilateral RCC as an adult with a strong family history of kidney cancer, likely all RCC. Unfortunately, our patient was not aware of the genetic status of family members. Although TSC is considered a hereditary kidney cancer syndrome, RCC is not in the diagnostic criteria and occurs in less than 5% of TSC patients [2, 4, 5, 13, 14, 20]. In fact, RCC may occur in as low as 1–2% of TSC patients, as older studies lacked the immunohistochemical markers to identify tumor lineage and therefore struggled to distinguish between epithelioid, fat-poor angiomyolipomas and renal epithelium-derived neoplasms [14]. Misdiagnosing renal angiomyolipomas as renal neoplasms in TSC patients may continue to be a modern-day issue [21].

RCC in TSC patients presents with varying, heterogenous histopathological appearances. The RCC in our patient had features that can be seen in renal epithelial neoplasms harboring mutations in *TSC1*, *TSC2*, *MTOR*, and *ELOC*(*TCEB1)* in addition to clear cell papillary renal cell tumor. Clinically, RCC in TSC patients frequently occurs bilaterally, more in women than men, and at younger ages than the general population [2, 5, 13, 14]. Interestingly, the RCC was unifocal and unilateral in our patient, and family history suggested unilateral RCC may have been common in the family members who had RCC. Additionally, our patient had the earliest known diagnosis of RCC in the family at 31-years-old. Although our patient tested negative for 19 genes known to cause renal cancer syndromes, including *FH*, *VHL*, *FLCN*, and *MET*, the possibility that the patient has a coexisting autosomal dominant renal cancer syndrome is unlikely but cannot be excluded.

Notably, since our patient had an adult presentation of RCC, history of uterine leiomyomas, and family history of RCC, we initially had reasonably high concern for HLRCC, another hereditary kidney cancer syndrome. It is presently unclear whether uterine leiomyomas can be a manifestation of TSC, which appears to be an understudied area of research [22, 23]. HLRCC is an autosomal dominant syndrome caused by pathogenic germline mutations in the *FH* gene. There is low penetrance of RCC in this syndrome, the median age of RCC diagnosis is 39–44 years old, and the RCC typically presents as a solitary, unilateral tumor [24]. Unlike in TSC-associated RCC, RCC in HLRCC is aggressive, high-grade, and associated with high mortality [24]. Current guidelines for HLRCC-associated RCC recommend prompt management with wide-margin partial nephrectomy due to the aggressive, infiltrative nature of the tumor [25]. Current guidelines for TSC-associated RCC, on the other hand, recommend active surveillance until the RCC reaches 3 cm followed by enucleation, as the tumor tends to be indolent in nature [25]. Therefore, genetic testing can be useful prior to intervention, if feasible.

Within six months of being diagnosed with RCC, our patient was diagnosed with PNET, another malignant tumor incidentally identified. Since PNET was initially reported so infrequently, there was debate over whether PNET was part of TSC clinical presentation [26]. It is now widely accepted that PNET is associated with TSC and included in TSC management guidelines [4]. However, there remains controversy over the frequency of PNET in TSC patients, as most are now identified incidentally; it is believed that less than 10% of TSC patients develop PNET [5, 15–19, 27]. One study observed that as low as 0.65% of TSC patients have nonfunctional PNET [27].

There are presently 61 cases of TSC-associated PNET reported in the literature, including this report, and are summarized in Table 1 [15–19, 27–51]. Two of the reports are non-English articles, limiting our ability to interpret the details [31, 33]. *TSC1* mutations were found in eight cases [18, 19, 27, 41]. *TSC2* mutations were found in 20 cases [16, 17, 19, 27, 34–37, 40, 44, 47, 51]. Nonfunctional PNET was found in 51 cases, 45 of which were found incidentally and the remaining six presented with abdominal/back pain secondary to mass effect [16–19, 27, 29, 34–41, 43–45, 47, 49]. Pediatric patients composed 26 cases [16–19, 27, 34, 35, 37, 39, 40, 51]. From Table 1, the median age of PNET diagnosis in TSC patients is 18 years old, which matches another analysis [27], and the average age of PNET diagnosis is 23 years old. In 2012, updated TSC surveillance guidelines recommended routine imaging for renal disease, which likely led to increased incidental PNET findings and in younger patients than seen prior to 2012 [52].

**Table 1:** Cases of PNET in TSC patients reported in the literature

Publication	Age of Diagnosis	Sex	TSC Genetics	History of Neurocognitive Dysfunction	Presentation	PNET	Location	Diameter	Histopathology	Management
Gutman et al. 1959 [28]	24 years	F	--	Convulsions and intellectual disability	Hypoglycemia	Insulinoma	--	3 cm	--	Surgical removal
Ilgren et al. 1984 [29]	23 years, deceased	F	--	Epilepsy	Incidental	Likely nonfunctional	--	--	--	Discovered during autopsy
Davoren et al. 1992 [30]	23 years	M	--	Seizures and mild intellectual disability	Hypoglycemia	Insulinoma	Head	3 cm	--	Surgical removal
Schwarzkopf et al. 1994 [31]^1^	34 years	M	--	--	--	Gastrinoma	Metastatic	--	--	--
Boubaddi et al. 1997 [33]^a^	18 years	F	--	--	Hypoglycemia	Insulinoma	--	--	--	--
Verhoef et al. 1999 [34]	12 years	M	*TSC2*, exon 13 C > T in position 1450/Q478X	Intellectual disability	Abdominal pain	Nonfunctional	Tail, metastatic to 3 lymph nodes	9.5 cm	--	Surgical removal
Eledrisi et al. 2002 [15]	43 years	M	--	--	Hypoglycemia	Insulinoma	--	21 cm	--	Surgical removal
Francalanci et al. 2003 [35]	6 years	M	*TSC2*, R1459X de novo mutation in exon 33	Epilepsy and intellectual disability	Incidental	Likely nonfunctional, claimed malignant	Tail	--	--	Surgical removal
Merritt et al. 2006 [36]	39 years	M	*TSC2*, 1 bp insertion at position 45–46	ADHD	Incidental	Likely nonfunctional	Numerous in body and tail	--	--	--
Sreenarsim-haiah et al. (2009) [49]	24 years	M	--	Seizures	Dull abdominal pain, weight loss	Nonfunctional	Head and uncinate process	4.8 cm	--	Surgical removal
Arva et al. 2012 [37]	15 years	M	*TSC2*, A > G in IVS17-2	No, but numerous tubers on MRI	Incidental	Likely nonfunctional	2 in body and tail and 4 micro-adenomas	11 cm, 1.2 cm, 1.5–5 mm	--	Surgical removal
Díaz et al. 2012 [38]	31 years	M	--	Seizures	Incidental	Nonfunctional	Tail, metastatic to 1/5 peri-pancreatic lymph node	2.3 cm	Well-differentiated, Grade 1, Ki-67 1%	Surgical removal
Larson et al. 2012 [16]	10 years	F	*TSC2*, exon 33 4308-11 frameshift	--	Incidental	Nonfunctional	Body/tail	1.2 cm	Well-differentiated, Grade 1	Surgical removal
	18 years	M	*TSC2*, exon 35 4646, missense GAP domain	--	Incidental	Nonfunctional	Body/tail	8 mm	Well-differentiated, Grade 1	Surgical removal; recurrence on imaging 33.8 months after resection [19]
	21 years	F	*TSC2*, exon 36, 4842-4, in-frame deletion GAP domain	--	Incidental	Nonfunctional	Body/tail	4.3 cm	Well-differentiated, Grade 2	Surgical removal
	39 years	M	--	--	Incidental	Nonfunctional	Body/tail	4.8 cm	Well-differentiated, Grade 1	Surgical removal
	48 years	M	--	--	Incidental	Nonfunctional	Body/tail	3.7 cm	Well-differentiated, Grade 1	Surgical removal
	51 years, deceased	F	--	--	Incidental	Nonfunctional	Body/tail	Micro-adenoma	Well-differentiated, Grade 1	Discovered during autopsy
van den Akker et al. (2012) [39]	Pediatric patient	M	--	--	Incidental	Likely nonfunctional	--	--	--	Surgical removal
Bombardieri et al. 2013 [40]	10 years	M	*TSC2*, c.5160 + 2_5160 + 3insT	Seizures and intellectual disability	Incidental	Nonfunctional	Isthmus	3.7 cm	Well-differentiated, Grade 2, Ki-67 4%	Surgical removal
Kang et al. 2017 [50]	23 years	M	--	Epilepsy and intellectual disability	Hypoglycemia	Insulinoma	2 in tail	3.5 cm, 7 mm	Well-differentiated, Grade 1, Ki-67 1.3% and 1.5%	Diazoxide followed by surgical removal
Koc et al. 2017 [17]	5 years	M	*TSC2* mutation	--	Incidental	Nonfunctional	Tail	2.6 cm	Well-differentiated, Grade 1	Decreased on everolimus followed by surgical removal
	12 years	M	--	--	Incidental	Nonfunctional	Tail	1 cm	Well-differentiated, Grade 2	Surgical removal
	13 years	M	--	--	Incidental	Nonfunctional	Tail	4 cm	--	Decreased on everolimus
	14 years	M	--	--	Incidental	Nonfunctional	Tail	2 mm	--	Observation with increase to 5 mm
	19 years	F	--	--	Incidental	Nonfunctional	Body	2.7 cm	--	Stable on everolimus
Comninos et al. 2018 [42]	67 years	F	--	Seizures after 47-years-old	Hypoglycemia	Insulinoma	Uncinate process	1.5 cm	Well-differentiated, Grade 1, Ki-67 1%	Subcutaneous octreotide and complex carbohydrate-rich diet followed by surgical removal
Mortaji et al. 2018 [41]	35 years	F	*TSC1*, c.1530_1531 delCA/ p.Asp510 Glufs*24	No	Incidental	Nonfunctional	Tail	1.1 cm	Well-differentiated, Grade 1, Ki-67 < 2%	Surgical removal
Amarjothi et al. 2019 [43]	17 years	F	--	No	Epigastric pain	Nonfunctional	Uncinate process	2.5 cm	Well-differentiated, Grade 1, Ki-67 2%	Surgical removal
Mehta et al. 2019 [18]	3 years	M	*TSC1*, exon 10 c.989dupT/ p.Ser331fs	Epilepsy	Incidental	Nonfunctional	Body	4 mm	Well-differentiated, Grade 2, Ki-67 ~ 15%	Increased to 1 cm in 16 weeks on observation followed by surgical removal
Reis et al. 2020 [44]	45 years	M	*TSC2*, deletion of exons 2–16 including initiation codon	Seizures and mild cognitive impairment	Incidental	Likely nonfunctional	Body, metastatic to liver	6 cm, largest met 7 cm	Low-grade	Deceased a few months after genetic evaluation
Al Qahtani et al. 2021 [46]	47 years	M	--	Epilepsy	Hypoglycemia	Insulinoma	Head	2 cm	Well-differentiated, Grade 1, Ki-67 < 2%	Surgical removal
Kopadze et al. 2021 [45]	34 years	F	Negative for *TSC1/2*; *COL4A4* p.(Gly774Arg) and p.(Gly1465Asp)	No, but several subependymal nodules and multiple cortical dysplasias by MRI	Incidental	Nonfunctional	Distal body/ proximal tail	1.6 cm	Suspected well-differentiated, Grade 1	Observation
Mowrey et al. 2021 [27]	3 years	F	*TSC1*, c.228 C > T	--	Incidental	Nonfunctional	Body	7 mm	--	Observation
	6 years	M	--	--	Incidental	Nonfunctional	Tail	2 cm	--	Surgical removal with history of mTOR inhibitor use
	7 years	M	*TSC1*, c.330insT	--	Incidental	Nonfunctional	Tail	1.2 cm	--	Surgical removal
	8 years	M	*TSC1*, 9 > 0insT12956	--	Incidental	Nonfunctional	Head	1 cm	--	Observation with history of mTOR inhibitor use
	9 years	F	--	--	Incidental	Nonfunctional	Tail	1.5 cm	--	Surgical removal
	9 years	F	*TSC2*, c.52,385,255 del18	--	Incidental	Nonfunctional	Head	1.5 cm	--	Observation with history of mTOR inhibitor use
	10 years	F	--	--	Incidental	Nonfunctional	Body	1 cm	--	Surgical removal with history of mTOR inhibitor use
	12 years	M	*TSC2*, c.4279delA	--	Incidental	Nonfunctional	Tail	1.7 cm	--	Surgical removal
	13 years	M	--	--	Incidental	Nonfunctional	Tail	1.4 cm	--	Observation with history of mTOR inhibitor use
	15 years	F	*TSC2*, c.3281 C > A	--	Incidental	Nonfunctional	Body	1.9 cm	--	Observation
	15 years	M	--	--	Incidental	Nonfunctional	Tail	2.3 cm	--	Surgical removal
	16 years	F	*TSC2*, 3 bp deletion of AAG	--	Incidental	Nonfunctional	Body	7 mm	--	Observation with history of mTOR inhibitor use
	18 years	M	*TSC2*, c.4646 A > G	--	Incidental	Nonfunctional	Body	1.1 cm	--	Surgical removal
	21 years	F	*TSC2*, 3 bp deletion of CAT, c.1108 C > T	--	Incidental	Nonfunctional	Body	4.1 cm	--	Surgical removal with history of mTOR inhibitor use
	32 years	M	--	--	Incidental	Nonfunctional	Head	3.8 cm	--	Unknown management, history of mTOR inhibitor use
	46 years	M	--	--	Incidental	Nonfunctional	Body	--	--	Surgical removal
Evans et al. 2022 [19]^b^	Pediatric patient	M	--	--	--	Nonfunctional	Body	1 cm	Well-differentiated, Grade 2	Surgical removal
	10 years	F	--	--	--	Nonfunctional	1 in neck, 1 in tail	2.0 cm, 2.4 cm	Well-differentiated, Grade 1	Surgical removal
	11 years	F	--	--	--	Nonfunctional	Tail	3.0 cm	Well-differentiated, Grade 2	Surgical removal
	18 years	F	--	--	--	Nonfunctional	Tail	3.8 cm	Well-differentiated, Grade 1	Surgical removal
	20 years	M	--	--	--	Nonfunctional	1 in body, 1 in tail	3.5 cm, 2.2 cm	--	Surgical removal
	30 years	M	--	--	--	Nonfunctional	Tail	5.1 cm	Well-differentiated, Grade 2	Surgical removal
	31 years	F	--	--	Incidental	Nonfunctional	Tail	9 mm	Well-differentiated, Grade 1	Surgical removal
	67 years	M	--	--	--	Nonfunctional	Tail	2.2 cm	Well-differentiated, Grade 1	Surgical removal
Piskinpasa et al. 2022 [48]	32 years	M	--	Seizures	Hypoglycemia	Insulinoma	Tail	1.2 cm	Well-differentiated, Grade 1, Ki-67 1%	Surgical removal
Zhang et al. 2022 [47]	43 years	F	*TSC2*, c.4700G > A; *BAP1*, c.1111dupA	--	Left back pain	Likely nonfunctional, FDG-avid	Body	2.5 cm	--	Surgical removal
Librandi et al. 2023 [51]	Pediatric patient	F	*TSC2*, c.1444-1G > A	Epilepsy	Seizure secondary to hypoglycemia	Insulinoma	Tail	2.9 cm	Well-differentiated, Grade 2, Ki-67 10%	Surgical removal
Present case	31 years	F	*TSC1*, 5’UTR deletion	No, but 2 tubers on MRI	Incidental	Nonfunctional	Body/tail	1.8 cm	Well-differentiated, Grade 1, Ki-67 < 1%	Surgical removal

-- denotes information not available

^a^Information limited due to reports written in languages other than English

^b^General cohort information about *TSC1/2* gene mutations and presenting symptoms are provided but not defined for specific patients and thus cannot be included in the table but are included in statistics in the discussion

Neurocognitive disorders, especially seizures and intellectual disabilities, have historically been seen as hallmarks of TSC due to Vogt’s triad, but four of the reported patients demonstrated no neurocognitive impairment despite limited data, suggesting TSC may present without Vogt’s triad more frequently than once believed [37, 41, 43, 45, 53]. One report from 1984 describes a TSC patient with epilepsy who may have had concurrent PNET and RCC, both of which were found on autopsy [29]. While many reports describe complex presentations in TSC patients with and without neurocognitive disorders, our report is the first in the literature to describe the co-occurrence of RCC and PNET in a patient with TSC in the absence of neurocognitive disorders. Moreover, given the unusually strong family history of renal disease, this particular *TSC1* mutation may have a strong propensity for kidney disease manifestation.

Management of TSC-associated PNET is an area of discussion. Currently, the National Comprehensive Cancer Network (NCCN) guidelines recommend surgical resection of PNET if symptomatic or > 2 cm in diameter and selective surgical resection of PNET that are 1–2 cm in diameter. However, it is not clear if TSC-associated PNET behave similarly to those seen in the average population. Notably, metastatic disease was found in four reported TSC-associated PNET cases, and one case had only a 2.3 cm PNET that demonstrated non-aggressive features on histopathology [31, 34, 38, 44]. Given the young age of our patient, surgical resection of the 1.8 cm PNET was discussed and agreed upon with the patient. In Table 1, PNET were surgically removed in 44 cases, of which only 12 cases were symptomatic, and clearly remains the standard for TSC-associated PNET management [15–18, 27, 28, 30, 34, 35, 37–43, 46–51]. However, the use of mTOR inhibitors may be an option for those who prefer observation [27].

With the growing number of *TSC1/2* variants and the wide spectrum of TSC penetrance and expressivity, it has been difficult to tease out genotype-phenotype correlations. Our patient had a 5’UTR deletion in the *TSC1* gene, which affects non-coding exon 1 and could contain the promoter region [54]. Analysis of *TSC1* gene mutations in families also suggested that exon 1 deletions are inactivating mutations and true null alleles [55]. Interestingly, the study also suggested that deletion of exon 1 could be related to an epilepsy phenotype, which we did not see in our patient [55]. When *TSC1* was inactivated in mouse models, the deletion was embryonic lethal for mice who inherited both inactivated genes and was lethal for a quarter of mice who inherited a single inactivated gene within the first couple days of birth [56]. Of those who inherited a single inactivated *TSC1* gene, 80% of mice developed solid RCCs by 15–18 months, sometimes resulting in grossly deformed kidneys, with some eventually metastasizing to the lungs [56]. This TSC mouse model exhibited the most aggressive phenotype compared to existing mouse models at the time and even compared to humans, making it unlikely to be an accurate reflection of human TSC pathology [56]. However, it remains unclear whether null alleles of *TSC1* in humans may result in a phenotype that generates several types of malignant tumors, potentially in the absence of more classic clinical manifestations of TSC.

Lastly, our patient was diagnosed with TSC late in life despite having a strong family history of TSC-associated clinical signs and symptoms and presenting with multiple clinical signs herself. The disparity in African American healthcare in the US, including the delay in their medical care, is well-known and strongly relates to social determinants of health as well as provider bias. However, TSC also presented unusually in this patient and, seemingly, in her family members, having exhibited scarce major clinical symptoms and an abnormally high rate of malignant cancers for TSC. Our report contributes to the published literature on the broad spectrum of disease seen in TSC.

## Conclusion

TSC is known for primarily forming benign tumors in any organ system with significant clinical consequences. TSC-associated RCC and PNET are independently rare manifestations of TSC. To our knowledge, this is the first report in the literature of TSC-associated RCC and PNET in one patient without neurocognitive dysfunction, which is also an unusually malignant phenotype for TSC. Both cancers were found incidentally by CT within six months of each other and were surgically removed out of concern for their malignant potential. TSC was diagnosed at the age of 31 years old in this patient with 5‘UTR deletion in the *TSC1* gene. Our patient had a strong family history of RCC and hypopigmented patches of skin, and the patient had a personal history of ungual fibromas of the toes, hypopigmented regions, and benign skin growths. MRI of the brain also identified two small, asymptomatic cortical tubers, one in each frontal lobe. There was no history of seizures or learning delay in the patient or her family. The incidental discovery and surgical management of RCC triggered this patient’s genetic evaluation, leading to a late diagnosis of TSC. Healthcare providers can be better informed of the broad spectrum of TSC presentation in patients and their families and have a low threshold to refer patients for genetic evaluation.

## Data Availability

All data underlying the results are available as part of the article and no additional source data are required. Additional information regarding the patient case is available from the corresponding author on reasonable request.

## Abbreviations

ADPKD: Autosomal dominant polycystic kidney disease
CT: Computed tomography
ED: Emergency department
EUS: Endoscopic ultrasound
FNA: Fine needle aspiration
HLRCC: Hereditary leiomyomatosis and renal cell carcinoma
MRCP: MRI magnetic resonance cholangiopancreatography
MRI: Magnetic resonance imaging
NCCN: National Comprehensive Cancer Network
PCKD: Polycystic kidney disease
PNET: Pancreatic neuroendocrine tumor
RCC: Renal cell carcinoma
TSC: Tuberous sclerosis complex
